# News and misinformation consumption: A temporal comparison across European countries

**DOI:** 10.1371/journal.pone.0302473

**Published:** 2024-05-08

**Authors:** Anees Baqir, Alessandro Galeazzi, Fabiana Zollo

**Affiliations:** 1 Department of Environmental Sciences, Informatics and Statistics, Ca’ Foscari University of Venice, Venice, Italy; 2 The New Institute Centre for Environmental Humanities, Venice, Italy; ’Enrico Fermi’ Research Center, ITALY

## Abstract

The Internet and social media have transformed the information landscape, democratizing content access and production. While making information easily accessible, these platforms can also act as channels for spreading misinformation, posing crucial societal challenges. To address this, understanding news consumption patterns and unraveling the complexities of the online information environment are essential. Previous studies highlight polarization and misinformation in online discussions, but many focus on specific topics or contexts, often overlooking comprehensive cross-country and cross-topic analyses. However, the dynamics of debates, misinformation prevalence, and the efficacy of countermeasures are intrinsically tied to socio-cultural contexts. This work aims to bridge this gap by exploring information consumption patterns across four European countries over three years. Analyzing the Twitter activity of news outlets in France, Germany, Italy, and the UK, this study seeks to shed light on how topics of European significance resonate across these nations and the role played by misinformation sources. The results spotlight that while reliable sources predominantly shape the information landscape, unreliable content persists across all countries and topics. Though most users favor trustworthy sources, a small percentage predominantly consumes content from questionable sources, with even fewer maintaining a mixed information diet. The cross-country comparison unravels disparities in audience overlap among news sources, the prevalence of misinformation, and the proportion of users relying on questionable sources. Such distinctions surface not only across countries but also within various topics. These insights underscore the pressing need for tailored studies, crucial in designing targeted and effective countermeasures against misinformation and extreme polarization in the digital space.

## Introduction

The advent of the Internet, followed by the rise of social media, has transformed access to information, empowering users to engage directly with content and receive real-time feedback. This shift has reshaped the information landscape, presenting both opportunities and challenges. A primary concern is the potential rapid dissemination of misinformation and its far-reaching impact on various aspects of society, spanning from the realm of politics [1–6], to critical societal issues like climate change [7] and vaccines [8–10]. The presence of misinformation on social media has been acknowledged as a phenomenon with the potential to influence the outcomes of crucial societal processes, leading scholars to increasingly focus on addressing this issue. As a response, extensive discussions involving scholars and policymakers have been centered on strategies to mitigate the spread of misinformation, including recent legislative initiatives within the European Union aimed at compelling social media platforms to implement countermeasures [11].

In recent years, many studies have been dedicated to understanding the dynamics and factors that may influence the spread of misinformation [12]. Some research works have compared the dissemination patterns of reliable and questionable content in various contexts, including science and conspiracy theories [13–15], the Covid-19 pandemic [16, 17], vaccines [10, 18], and elections [6], revealing differences in diffusion dynamics and prominence between reliable and unreliable news sources. Researchers have also investigated the role of the information environment in the spread of misinformation, underscoring how polarized debates can create fertile ground for its dissemination [19]. Echo chambers, where like-minded individuals reinforce their beliefs through repeated interactions, have been explored, indicating that misinformation primarily circulates within specific user groups [20]. Furthermore, factors suspected of influencing news consumption may include social media recommendation algorithms and users’ selection bias, which can influence the user’s exposure to ideologically diverse news [21–24], and automated accounts, which have been implicated in amplifying misinformation [1, 25, 26]. Furthermore, social and cultural factors may influence the way topics are perceived and the efficacy of countermeasures to limit the impact of misinformation [27, 28].

While there exists a significant amount of literature addressing misinformation, only some studies examine its impact over time and across different countries [10, 29] or topics [30–32]. However, to our knowledge, there has been no simultaneous investigation into all dimensions. Still, countries may exhibit different levels of sensibility and resilience to misinformation [33]. This may depend on different factors, from intrinsic cultural variables to topic-dependent characteristics. Thus, studying how topics are perceived across countries is essential to limit the spread of misinformation and design tailored counter-strategies [27]. Indeed, understanding the dynamics of consumption and the magnitude of diffusion of reliable and untrustworthy news in social media, as well as the characteristics of the communities consuming them, can provide valuable insights into designing appropriate countermeasures to reduce the impact of unfounded news. Policies need to be tailored to the social context where they will be deployed [34].

In this work, we took a distinct approach by conducting a comparative analysis of misinformation spreading and consumption spanning various topics in diverse European countries. This approach enabled us to highlight the differences and similarities in interest, engagement, and consumption of information over time and across European countries, thus emphasizing the features of misinformation in each debate. Specifically, our aim is to tackle the following research questions: RQ1) Do consumption patterns of reliable and questionable news vary depending on the country and topic being considered? RQ2) To what extent are reliable and questionable sources segregated within the information landscape, and how diverse is the users’ information diet? RQ3) Do questionable and reliable communities remain consistent over time and across different topics? To address these questions, we investigated the consumption of Twitter content produced by news outlets in Europe, focusing on events from 2019 to 2022. Our goal was to offer a comparative assessment of the information landscape across multiple countries. To ensure a topic-independent analysis, we select one subject per year that has been debated in all four countries under consideration: France, Germany, Italy, and the United Kingdom. We focused solely on these countries because of the limited data availability regarding the reliability of news outlets. However, it is worth noting that these four countries encompass the four most populous nations in Western Europe.

We analyzed the engagement generated within these countries and around these topics while also considering the reliability of the content sources. For this assessment, we used the classification provided by NewsGuard. This classification is conducted by independent professionals based on nine journalistic criteria (refer to section Materials and methods for details), which remain consistent across all selected countries, ensuring a homogeneous comparison. Furthermore, we constructed similarity networks based on the consumption patterns of news outlets’ content, allowing us to compare the diverse structures that emerge across countries and topics.

Our findings revealed that reliable sources dominated the information landscape, though there was active participation from questionable user groups in the debate. Notably, our networks indicated that some users engage with both types of information sources. Furthermore, our cross-country comparison uncovered variations in the similarity structure of news sources among countries, ranging from a clear separation of questionable sources to a more mixed composition with no significant differences.

Overall, our results highlighted disparities as well as commonalities in news consumption among the chosen countries, especially concerning subjects of shared European interest, offering a valuable view of the topic perception across different European nations. We also emphasized the role played by questionable sources, providing insights at both the country and topic levels that can be leveraged in the design of effective measures to counter misinformation.

## Materials and methods

### Data collection and processing

Twitter data have been downloaded using the official Twitter API for academic research (see https://developer.twitter.com/en/docs/twitter-api), freely available for academics at the time of collection, and analyzed in compliance with the Terms and Conditions of Twitter(now X). Based on the list of accounts retrieved from the NewsGuard dataset (see Table 1), we downloaded the Twitter timelines of media sources based in Italy, Germany, France, and the UK over three years from 2019 to 2021. NewsGuard(https://www.newsguardtech.com/solutions/newsguard/) is a tool that evaluates the reliability of news outlets based on nine journalistic criteria. Following such criteria, a team of professional and independent journalists assigns a “trust score” between 0 and 100 to each news outlet. Ratings are not provided for individuals, satirical content, or social media platforms like Twitter, Facebook, and YouTube. News sources are categorized into two groups based on their score: Reliable (trust score greater or equal to 60) and Questionable (trust score less than 60). The threshold is set by NewsGuard based on the evaluation criteria. The NewsGuard coverage of classified news outlets varies by country, yet it consistently includes a sufficient number of sources to represent at least 90% of the engagement in each respective nation.

**Table 1:** Breakdown of the NewsGuard news sources dataset by country and reliability.

Country	Reliable sources	Questionable sources	Total
France	187	49	236
Germany	196	25	221
Italy	175	29	204
UK	191	22	213
Total	749	125	874

We collected only publicly available content from public Twitter accounts rated by NewsGuard. The dataset included all the tweets published by the selected accounts in the period from 01 January 2019 to 11 November 2021, resulting in 25+ Million tweets. Table 2 reports the breakdown of the data. The percentage of posts by each country contributing to the total amount is shown in parentheses.

**Table 2:** Volume of tweets by country and reliability.

Country	Number of tweets	Reliable tweets	Questionable tweets
France	7,083,659 (28.19%)	6,151,554 (26.57%)	932,105 (47.32%)
Germany	4,904,179 (19.52%)	4,689,186 (20.25%)	214,993 (10.91%)
Italy	4,936,407 (19.65%)	4,528,606 (19.56%)	407,801 (20.70%)
UK	8,201,352 (32.64%)	7,786,239 (33.62%)	415,113 (21.07%)
Total	25,125,597	23,155,585	1,970,012

To ensure that our analysis concentrated on topics debated at the European level for cross-country comparisons, we applied keyword filters to our original dataset based on the topic modeling results (see paragraph Topic Modeling. We divided our dataset into three one-year segments and filtered each segment according to a list of keywords related to the most discussed topic at the European level for that year identified using topic modeling (see paragraph Topic Modeling). The statistics for the filtered data can be found in Table 3. For the tweets in the filtered dataset, we collected all retweets. Details about the number of original tweets and retweets for each topic can be found in Table 3.

**Table 3:** Breakdown of the filtered dataset by country and topic.

	Keywords		France	Germany	Italy	UK	Total
**Brexit**	brexit	Users	33,288	22,512	8,676	231,911	296,387
		News sources	129	127	89	167	512
		Tweets	12,493	6,368	3,877	46,404	69,142
		Retweets	97,909	53,352	24,856	1,385,023	1,561,140
**Coronavirus**	ncov, corona*, covid*, sars-cov-2	Users	461,737	541,773	146,205	910,955	1,940,670
		News sources	218	192	171	202	783
		Tweets	204,728	99,527	117,899	137,913	560,067
		Retweets	3,548,617	2,270,063	1,474,654	3,613,294	10,906,628
**Covid Vaccine**	vacc*, astrazeneca, vaccin*, moderna, pfizer, sinopharm, sputnik, biontech	Users	396,131	165,122	156,273	303,365	1,020,891
		News sources	214	180	171	192	757
		Tweets	133,962	37,721	136,814	44,212	352,709
		Retweets	2,630,179	779,521	1,943,585	1,099,068	6,452,353

The data utilized in this study, shared in accordance with the terms and conditions of the sources, can be accessed at https://osf.io/q7h8u/

### Topic modeling

We utilized BERTopic, a topic modeling tool that extracts latent topics from a collection of documents, to identify the heated topics prevalent in all the countries under examination. BERTopic is a top2vec model generalized for pretrained sentence transformers [35] that has recently demonstrated promising results in various tasks. BERTopic generates coherent clusters of documents through three steps: 1) extracting document embeddings; 2) clustering embeddings; 3) creating topic representations using class-based TF-IDF [36] (c-TF-IDF). In the first step, any pre-trained transformer-based language models can be utilized, allowing the use of state-of-the-art embedding techniques. The second step employs uniform manifold approximation and projection (UMAP) to reduce the dimension of embeddings [37], and hierarchical density-based spatial clustering of applications with Noise (HDBSCAN) to generate semantically similar clusters of documents [38]. One of the topics is set to be ‘others’, and includes the documents that are not included in different topics.

### Similarity networks

We assessed the audiences’ similarity among news outlets exploiting the retweets of the content they produced. For each country and topic, we built an undirected weighted graph *G*, in which nodes represent news outlets and edges the audience similarity among them. We started by creating a matrix *R*_*c*,*t*_ for each country and each topic, with retweeters as rows and news outlets as a column, whereas *c* ∈ *{France, Germany, Italy, UK}* and *t* ∈ *{Brexit, Coronavirus, Covid Vaccine}*. The entry *r*_*i*,*j*_ of *R*_*c*,*t*_ is the number of times user *i* retweeted a tweet posted by news source *j* based in the country *c* on topic *t*. We then computed the cosine similarity for each pair of columns to measure the audiences’ similarity for each pair of news sources. Thus, the weight *w*_*a*,*b*_ of the edge between node *a* and *b* in the graph *G* is equal to:

$$\begin{array}{c} w_{a,b}=\frac{r_{a}\cdot r_{b}}{∥r_{a}∥∥r_{b}∥} \end{array}$$

where *r*_*a*_ and *r*_*b*_ are the two column vectors of news sources *i* and *j*, respectively. It should be noted that *w*_*a*,*b*_ ∈ [0, 1] since all the entries of the matrix are non-negative. Thus, news outlets that have a substantial overlap in retweeters tend to have a similarity value approaching 1, whereas outlets with minimal shared retweeters result in a lower similarity (nearing 0).

Finally, we excluded all the 0-degree nodes and deleted all the edges with a weight below the median of all edge weights. This approach enabled us to capture the strongest similarities among news outlets’ audiences related to the selected topics within the European context.

## Results and discussion

In this section, we present the results of our analysis, organized as follows. First, we provide an overview of the information landscape in selected European countries over the three years. This step is crucial for identifying key topics that are widely shared among countries and distinguishing between questionable and reliable sources, enabling a coherent comparison. We recall that we limited our analysis to these countries due to the limited availability of NewsGuard ratings within this specific subset of European countries. Next, we examine both commonalities and differences among countries in their online discussions of these topics, focusing on user engagement and consumption patterns.

### The evolution of public discourse across countries

**Fig 1:**
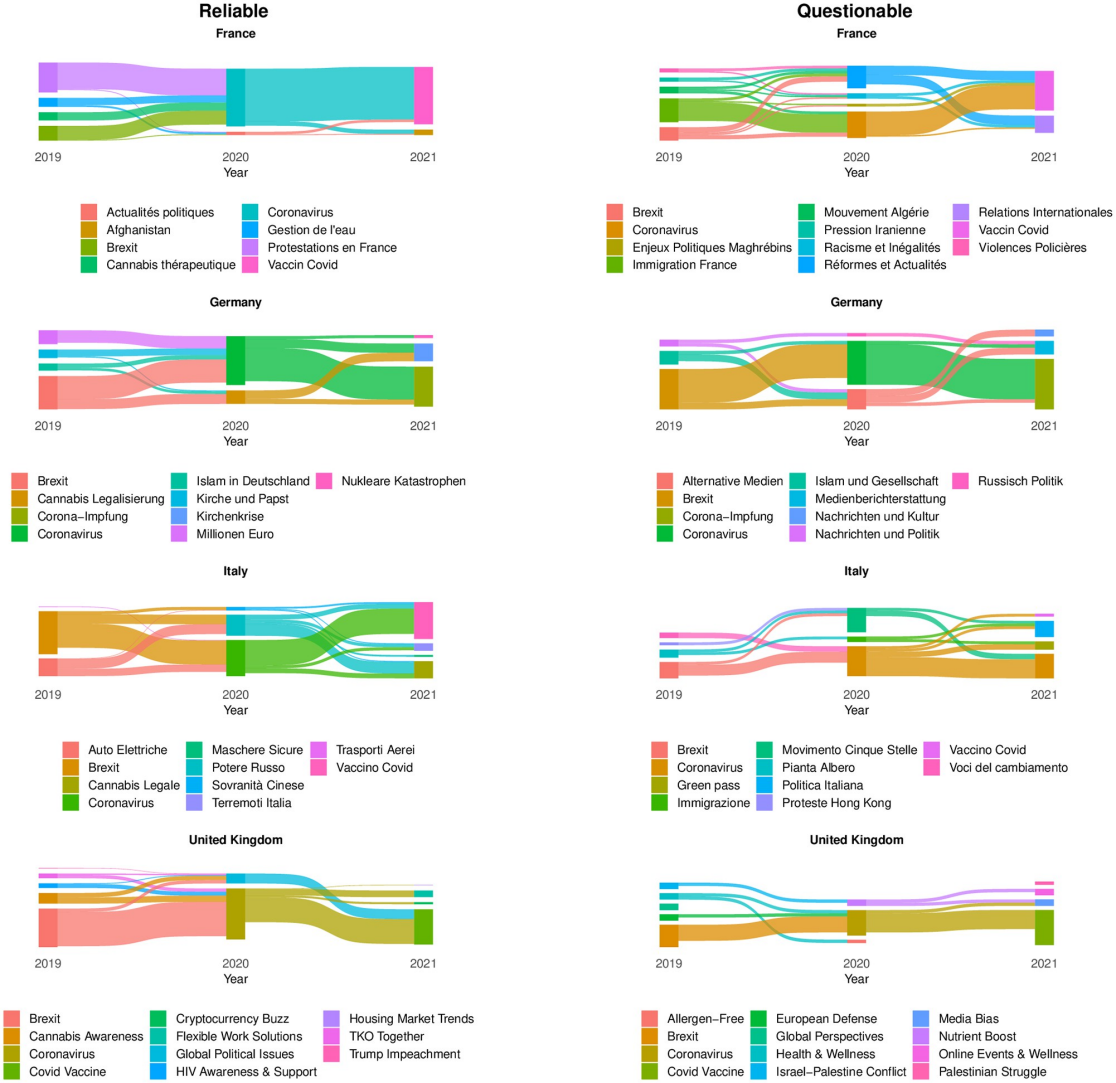
Topic modeling results on questionable and reliable news sources’ content across countries. The size of each topic is given by the proportion of unique news sources contributing to it. The flows represent the interest shift of news outlets in different topics over time, and the size of each topic across years is proportional to the number of news sources discussing that topic.

To compare the landscapes of public discourse in the selected countries, our initial step involves identifying common topics extensively discussed in all four countries and by both questionable and reliable sources. To this aim, we employ BERTopic [35] to perform topic modeling on the content produced by news outlets’ accounts over three years (see Section Materials and methods for further details). To identify suitable topics for our analysis, we divide the dataset by year and by country and run BERTopic algorithm on each subset. The results reported in Fig 1 show the most debated topics for each year by country and source category. The size of each topic represents the number of news sources contributing to it, while its position reflects its relevance to the overarching topics. The flow diagrams show the topic’s prevalence in news outlets over time. In particular, the flow connecting two topics illustrates the evolving focus of news sources over time. It represents the number of sources generating content about a specific topic as it shifts from one subject to another. The width of the flows indicates the varying presence of sources in the ongoing debate.

Fig 1 highlights how the attention of news outlets to different topics varied across countries and types of news sources. Notably, in addition to certain topics of common interest, news outlets tended to prioritize subjects of national relevance, such as protests, the influence of foreign countries, religion, electric cars, and drug legalization. We also observe disparities in the topics covered by questionable and reliable sources within the same country. For instance, the fraction of news outlets reporting on the coronavirus vaccine in Italy was higher for reliable sources than for questionable ones. Furthermore, certain topics were exclusive to one type of source, like “Flights” (Italy, reliable), “Water management” (France, reliable), or “Palestinian struggle” (UK, questionable). These findings indicate that the level of interest was influenced both by the country and the type of source considered, with questionable sources displaying a broader range of interests and reliable ones focusing more on topics common to all countries.

Crucially, our analysis highlights the presence of common topics between both questionable and reliable debates of all countries. Specifically, three topics appeared consistently in debates across all countries: “Brexit”(2019), “Coronavirus”(2020), and “Covid Vaccine”(2021). Therefore, in the subsequent analysis, we exclusively focus on these topics for a cross-country examination of the discourse. The rationale behind this choice is to spotlight the differences and similarities in how these topics were reported and consumed by news outlets and users from various countries, thereby minimizing the impact of topic-specific variations on our analysis. Additionally, these topics have been extensively discussed at the European level, making our analysis valuable for understanding how subjects of European significance are perceived across different countries.

To underscore the relevance of the three chosen topics in online public debates and validate the accuracy of the time frames assigned to each topic, we conduct a Google Trends analysis of search interest in Brexit, Coronavirus, and Covid Vaccine in France, Germany, Italy, and the UK from 2019 to 2021, as shown in S11 Fig.

The analysis of Google Trends confirms that the selected topics attracted the highest attention during the specified time frames in the broader online context. Thus, going forward, our analysis focuses on these three topics (Brexit, Coronavirus, and Covid Vaccine) to examine the differences and similarities in news production and consumption within the European landscape. To conduct our analysis exclusively on these topics, we filter the timelines of news outlets to select only tweets relevant to the chosen topic within the respective time range (see Section Materials and methods for details).

### User engagement across topics and countries

**Fig 2:**
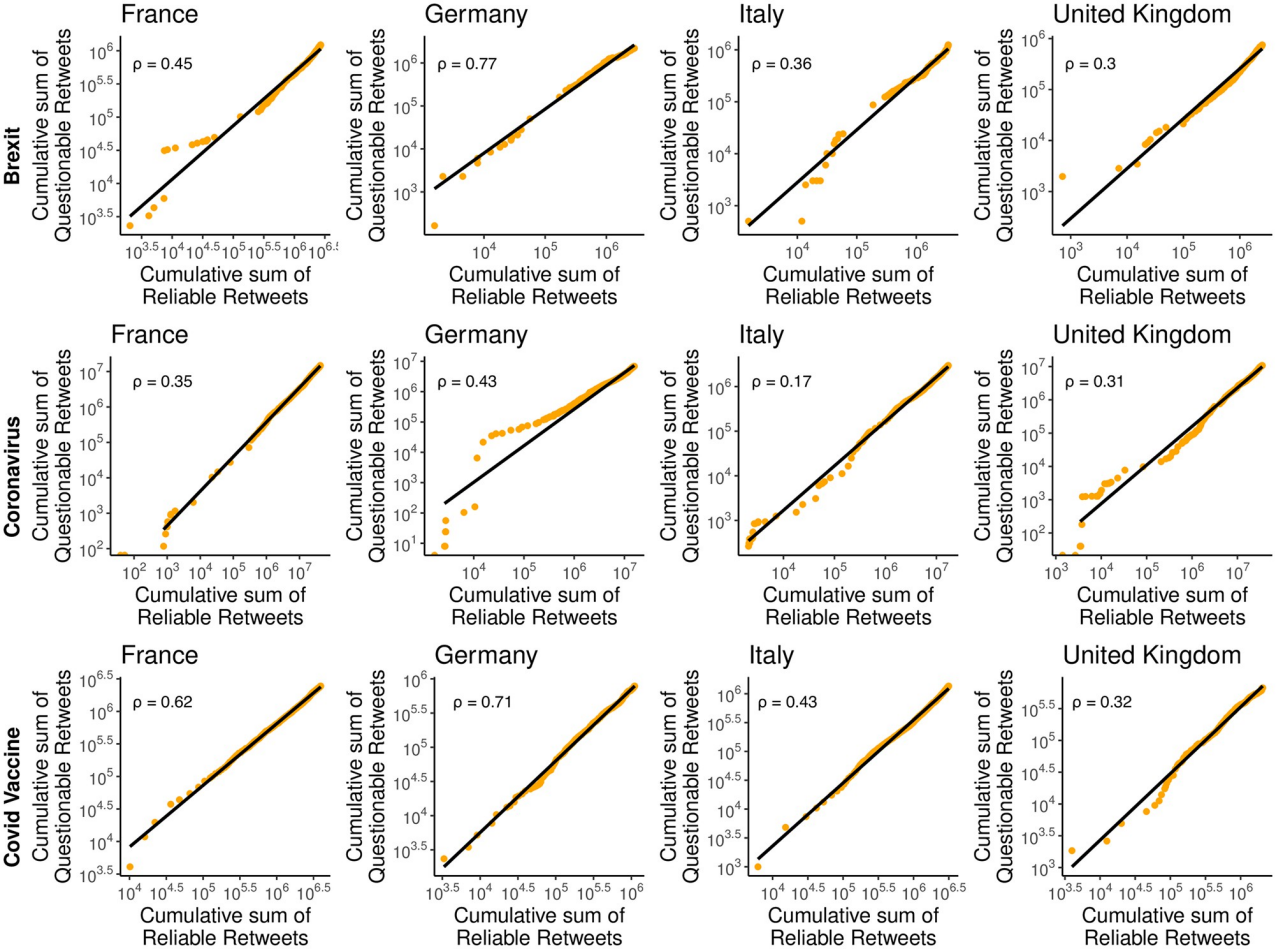
Cumulative number of the total retweets received by questionable sources vs the cumulative number of retweets for reliable sources across different countries and topics. The x and y coordinates of each dot represent the cumulative number of retweets received by reliable and questionable sources respectively. This cumulative number is calculated by arranging the retweets based on their creation time and aggregating them on a daily basis. A linear trend suggests that the temporal patterns of questionable engagements mirror a scaled version of reliable engagements. Each graph displays the *ρ* coefficient, illustrating the ratio of volumes between retweets received by questionable and reliable posts.

To address RQ1, we continue our study by comparing engagement with content related to the identified topics on social media platforms. Fig 2 illustrates the cumulative distribution of retweets for questionable sources compared to the cumulative distribution of retweets for reliable sources across different countries and topics (the classification of the sources is based on NewsGuard data, as detailed in Section Materials and methods). We specifically focus on retweets to maintain coherence with the rest of the analysis, which employs retweets to study users’ interactions (the same analysis for likes, quotes, and replies can be found in S8–S10 Figs).

The observed linear relationship between questionable and reliable sources suggests a similar spreading dynamic. The presence of dots not aligning with the fitted slopes may be due to the occurrence of posts going viral, leading to a dramatic increase in retweets for one of the two categories. However, these outliers are rare, as confirmed by the accuracy of the linear fits (see Supporting information for more details on the linear fits). Additionally, we observe that across all countries and topics, the ratio *ρ* between the total number of questionable and reliable retweets is consistently lower than 1, indicating that reliable sources, overall, attracted more attention than questionable ones, regardless of the topic and country under consideration.

However, France, Germany, and Italy experienced a significantly higher share of questionable retweets in Brexit and Covid Vaccine debates compared to the Coronavirus discussion. In contrast, the United Kingdom maintained a relatively stable proportion between questionable and reliable retweets across different topics. Differences also emerge when comparing the questionable vs reliable retweets ratio across countries, with Germany having the highest ratio for all topics, followed by France. Italy ranks third in all cases except for Coronavirus, while the United Kingdom has the lowest fraction of questionable retweets in all cases except for this topic.

To offer a complete view of the varying levels of engagement received by questionable sources, we provide the distribution of the number of retweets, likes, replies, and quotes counts for questionable and reliable sources in S4–S7 Figs, respectively, for each country and topic considered. Overall, the results highlight the dependency of questionable content consumption on both the topic and the country in question, showing a significant variation in the share of engagement received by questionable sources.

### News outlets similarity and users’ diet

**Fig 3:**
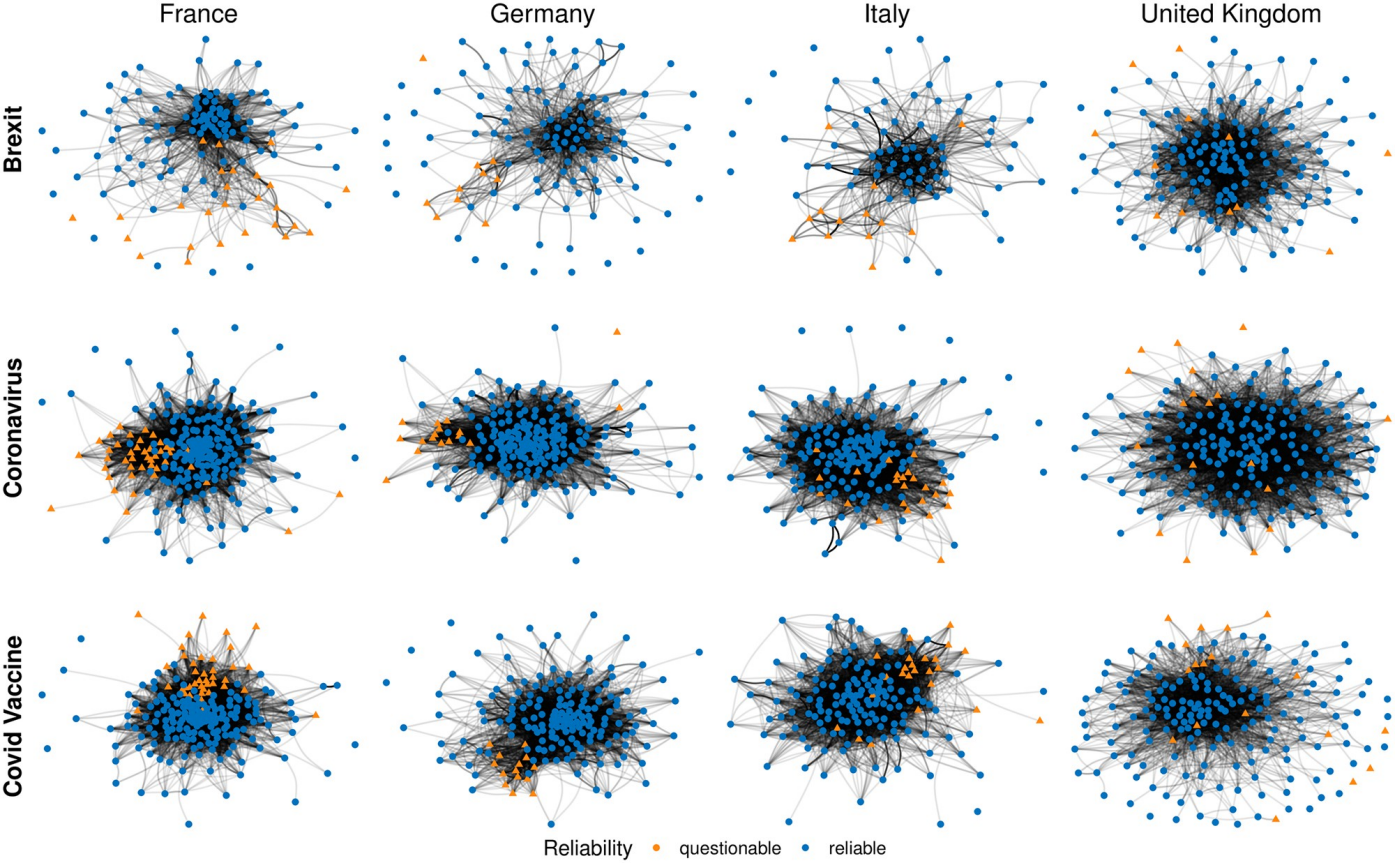
Similarity network among news outlets, where each news source is represented as a node, and edges represent audiences’ similarity among news outlets. The color and shape of the nodes indicate the classification of the news source, and the thickness of the edges represents the level of similarity of retweeters between two news sources. We discarded edges with weights lower than the overall median of the edges. Each network represents the news outlets’ similarity on one topic for one country. Networks are represented using the Fruchterman-Reingold force-directed graph drawing algorithm.

To answer RQ2, we turn our attention to news consumption patterns and study the interplay between reliable and questionable sources, as well as the variety of users’ news diets. Analyzing Twitter data on Brexit, Coronavirus, and Covid Vaccine, we first explore whether news outlets of the same type are consumed (i.e. retweeted) by similar audiences. We define a metric based on cosine similarity(see Section Materials and methods) on retweeters to quantify the similarity between news outlets in terms of audiences. News outlets sharing a high percentage of retweeters have a higher value of the similarity metric (close to 1), while outlets with only a few shared retweeters get a low similarity (close to 0). We then build an undirected network in which news outlets are represented as nodes and weighted edges indicate the level of similarity among them. We create one network for each country and topic considered to enable a fair comparison. The resulting networks are visualized in Fig 3. To highlight only the stronger connections, we discard edges with weights lower than the overall median of the edges of each network (see S1 and S2 Figs of SI for the results with the complete networks).

We may observe variations in the network structure depending on the country and topic under consideration. Indeed, France, Germany, and Italy tend to display a clearly identifiable cluster of questionable sources (orange triangles), indicating the presence of communities primarily consuming questionable content. In the UK, this distinction is less pronounced. Looking at topic-specific differences, we find that for all countries except the UK, the networks tend to be sparser, with a lower edge density, in the case of Brexit. For Coronavirus and Covid Vaccine discussions, the networks are more connected and exhibit higher edge density (see Table S2 of S1 File). This is reflected in the separation between questionable and reliable news sources: in the Brexit debate, the separation between the two types of news appears clearer, while in the other debates, they share a higher number of connections, as shown in Table S3 of S1 File. To quantify this behavior further, we apply the adjusted nominal assortativity to our networks. We chose this measure to account for the imbalance between the number of questionable and reliable news, which may confound traditional measures used to quantify segregation in networks [39]. The results indicate that higher levels of assortativity are observed in the context of the Brexit debate for all countries except the UK, which exhibits different behavior possibly attributed to its direct involvement in the discussion (see Table S1 of S1 File).

**Fig 4:**
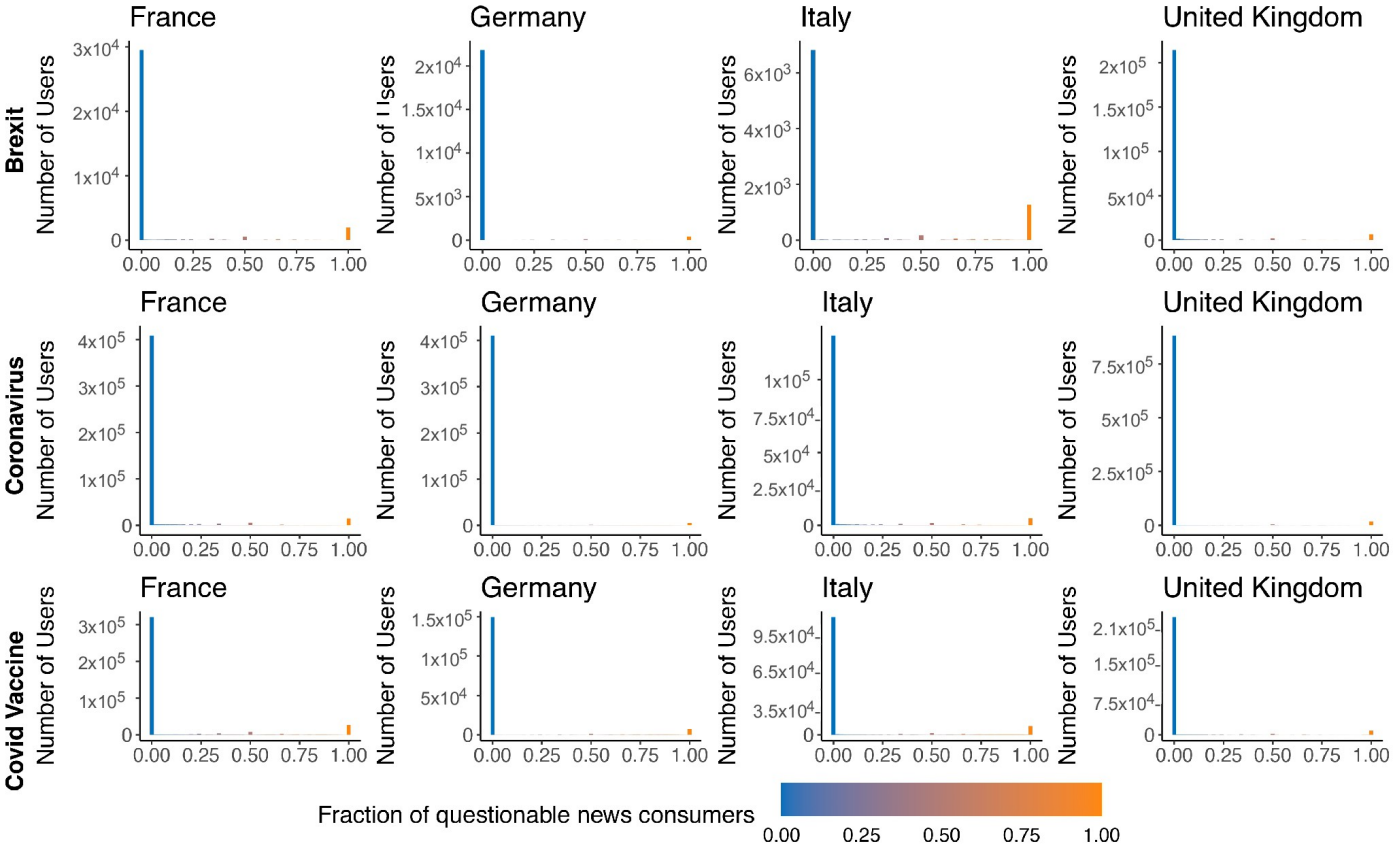
Analysis of users’ consumption behavior through retweets. Each histogram represents the user count versus the fraction of news from questionable sources, ranging from entirely reliable (0) to entirely questionable (1). A dominant presence near lower fractions suggests a prevalent reliance on reliable sources. In contrast, significant increases near the higher end highlight segments influenced by questionable content.

Our analysis also reveals that there is no absolute separation between questionable and reliable news outlets. This suggests that some users primarily or exclusively consume reliable or questionable content, while others have a mixed news diet, consuming both types in varying proportions. To delve deeper into this question, we analyze the fraction of questionable news consumed by each user and present the distribution in Fig 4. The results indicate that the majority of users in each debate primarily rely on reliable news sources (see also Table S4 of S1 File). However, in every debate, there is a small but noticeable fraction of users who exclusively endorse questionable news, although with varying degrees of prominence. Notably, the figure depicts a distinctive bimodal distribution, with few users falling outside the extreme ends of the spectrum. These users play a crucial role in bridging the gap between questionable and reliable news within the similarity networks. Furthermore, reliable news sources tend to occupy the core of the network, while questionable sources are generally situated in more peripheral positions. Indeed, among the top 25 sources identified by the PageRank algorithm in each network [40], a substantial majority (at least 95.3%) are found to be reliable news sources (see Supporting information for further details).

### Community structure and users’ persistence

We conclude our analysis by examining the community structure within the similarity network and exploring user persistence in retweeting news outlets across topics, addressing RQ3. This examination seeks to identify the presence of distinct communities among news outlets within similarity networks, as well as the extent to which users consistently engage with the same sources across different topics.

**Fig 5:**
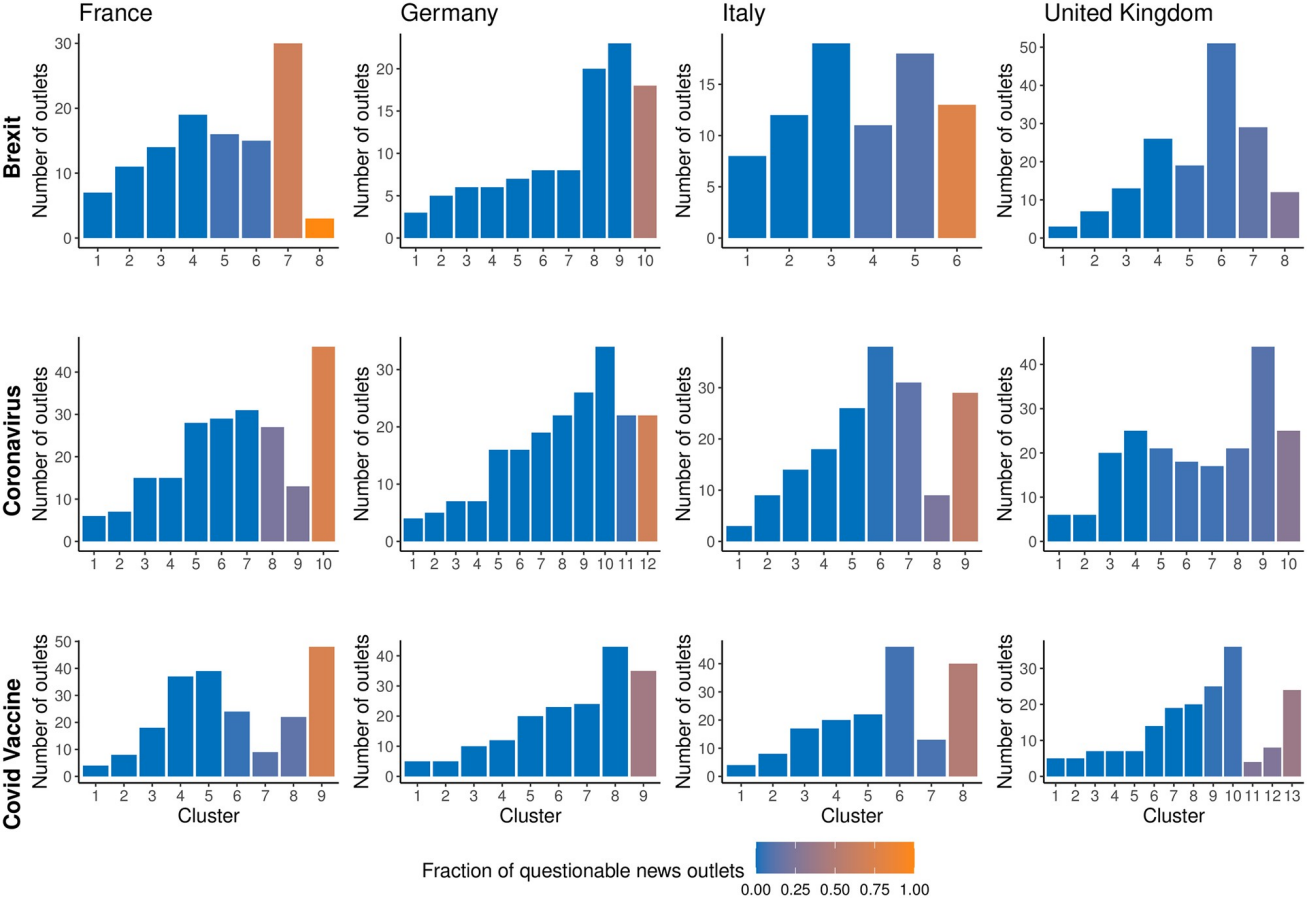
Community detection analysis of news outlets’ similarity networks. Clusters were found using the Louvain clustering algorithm and sorted based on the percentage of questionable news outlets. The percentage of questionable sources in each cluster is color coded. Network edges with weights lower than the median value were discarded here, result with the complete network is reported in SI.

Thus, we perform community detection using the Louvain clustering algorithm [41] and report the results in Fig 5. Clusters are color-coded based on the proportion of questionable news outlets, with darker shades indicating a higher percentage of questionable sources.

Across all countries and topics, the majority of clusters consisted mainly of reliable news outlets, and within these clusters, we also find the most significant nodes according to the PageRank classification. However, our analysis also reveals the presence of small clusters with a high proportion of questionable news outlets. The number and size of these clusters vary depending on the country and topic. For instance, in Germany and Italy, there is one such cluster for each topic, while in the Brexit debate in France, there are two clusters. In the UK, the separation is less clear, with no clusters showing a high percentage of questionable news outlets. We also notice that reliable clusters tend to be smaller in size but more numerous, while questionable clusters tend to be larger and often unique in each network. This suggested that users who consume questionable content tend to endorse most of the questionable sources of the network, while reliable news consumers focus on fewer news outlets.

We also investigate the persistence of users in consuming questionable and reliable news. To do this, we compute the Jaccard similarity across topics for retweeters of questionable and reliable sources, as shown in Table S5 of S1 File. Results indicate a low Jaccard score for all pairs of topics and countries, suggesting that most users have not retweeted the same sources in all debates. However, questionable news sources experienced slightly higher users’ persistence in the case of Coronavirus—Covid Vaccines compared to reliable news outlets.

Additionally, we analyze whether groups of users retweeting questionable content with highly similar consumption behavior are comprised of bots. We selected all users whose diet consists of questionable content for more than half, calculating pairwise cosine similarity. Subsequently, we consider only users with a similarity score higher than 0.5 and use Botometer (https://botometer.osome.iu.edu/) to assess their likelihood of being automated accounts. Among these users, only 1.4% of them are identified as bots (botometer score higher than 3), and 4.2% are close to being classified as bots (botometer score between 2 and 2.5).

Overall, our analysis provides a longitudinal view of the online news consumption landscape in the selected countries, highlighting the predominance of reliable news sources while also revealing the presence of clusters with a higher proportion of questionable news sources in many countries and topics. The existence of such clusters suggests the presence of a group of users consuming content from various questionable sources while avoiding reliable ones. This behavior is consistent with the potential presence of echo chambers, a phenomenon widely observed in online debates [7, 20, 42].

## Conclusion

In this study, we have delved into the evolving dynamics of news production and consumption within the European context. We examined the consumption of Twitter content produced by news outlets in France, Germany, Italy, and the United Kingdom, providing a cross-country and cross-topic comparison of the online public discourse. We identified topics debated across all four countries and highlighted differences and similarities in consumption patterns. Additionally, we constructed networks based on the similarities among news outlets’ audiences, revealing the presence of groups of users engaging with sources of different reliability.

Our findings indicated that reliable sources dominate the information landscape, but users consuming content mainly or exclusively from questionable news outlets were often present. However, the size and importance of such groups vary based on the topic and the country under consideration. Furthermore, our cross-country comparison has revealed variations in the structure of news sources’ similarity networks. While some countries exhibited a clearer separation between clusters of questionable sources and reliable sources, others showed a more heterogeneous situation with less detectable differences in cluster composition. However, the connectedness of the networks and users’ behavior analysis indicated the presence of a small fraction of users with a mixed news diet in all countries.

Our results emphasized the differences and similarities in news consumption patterns across countries in relation to globally significant subjects. However, the multitude of factors contributing to variations among countries and topics suggests a rich and complex landscape for exploration. Future studies should aim to investigate the underlying causes driving these phenomena, with particular focus on factors that might contribute to increasing the engagement of misinformation sources. Understanding the dynamic of news consumption and its dependence on factors such as the topic or country can provide valuable insights into the development of effective countermeasures to mitigate the spread of misinformation and disinformation. Monitoring the information landscape at both national and European levels is indeed crucial to understanding the state of public discourse on contentious topics and and detecting the emergence of new and divisive narratives within the European context. Also, it is essential to recognize that effective policies may require tailoring to specific cultural settings and differences. Insights gained from one country or setting may not necessarily be universally valid or applicable in others. Therefore, developing tailored, cohesive strategies to improve the health of information ecosystems at the European level must take into account these cultural nuances and differences.

## Supporting information

S1 Fig Similarity network among news outlets. Each news source is represented as a node, and edges represent audiences’ similarity among news outlets. The color and shape of the nodes indicate the classification of the news source, and the thickness of the edges represents the level of similarity of retweeters between two news sources. Each network represents the news outlets’ similarity on one topic for one country.
(TIFF)

S2 Fig Community detection analysis of news outlets’ similarity networks with all the edges. Clusters were found using the Louvain clustering algorithm and sorted based on the percentage of questionable news outlets. The percentage of questionable sources in each cluster is color coded.
(TIFF)

S3 Fig Distribution of News Outlets type respect to PageRank score. The distribution shows the dominance of reliable sources (blue) over questionable sources (orange).
(TIFF)

S4 Fig Distribution of retweets by country for reliable (blue) and questionable (orange) news sources around Brexit (top row), Coronavirus (middle row), and Covid Vaccine (bottom row). (TIFF)

S5 Fig Distribution of likes by country for reliable (blue) and questionable (orange) news sources around Brexit (top row), Coronavirus (middle row), and Covid Vaccine (bottom row). (TIFF)

S6 Fig Distribution of replies by country for reliable (blue) and questionable (orange) news sources around Brexit (top row), Coronavirus (middle row), and Covid Vaccine (bottom row). (TIFF)

S7 Fig Distribution of quotes by country for reliable (blue) and questionable (orange) news sources around Brexit (top row), Coronavirus (middle row), and Covid Vaccine (bottom row). (TIFF)

S8 Fig Cumulative number of the the total likes received by questionable sources vs the cumulative number of likes for reliable sources across different countries and topics. (TIFF)

S9 Fig Cumulative number of the the total quotes received by questionable sources vs the cumulative number of quotes for reliable sources across different countries and topics. (TIFF)

S10 Fig Cumulative number of the the total replies received by questionable sources vs the cumulative number of replies for reliable sources across different countries and topics. (TIFF)

S11 Fig Google Trends analysis of search interest in Brexit, Coronavirus, and Covid Vaccine in France, Germany, Italy, and UK from 2019 to 2021. The plots display how search interest for each topic evolved over time, with each row representing one topic. Interest trends reveal that Brexit was most popular in 2019, followed by a sharp decline in 2020 and 2021 with some exceptions at the end of 2020. Coronavirus peaked in early 2020 and declined thereafter, while Covid Vaccine gained momentum in early 2021, reached the maximum in mid-2021, and saw another surge at the end of 2021. Brackets represent the time span taken into account in the analysis for each topic.
(TIFF)

S1 File (PDF)
