# Supplementary figures and images for: News and misinformation consumption: A temporal comparison across European countries
Source: PLoS One. 2024 May 8;19(5):e0302473. doi: 10.1371/journal.pone.0302473 (PMC11078435; doi:10.1371/journal.pone.0302473)

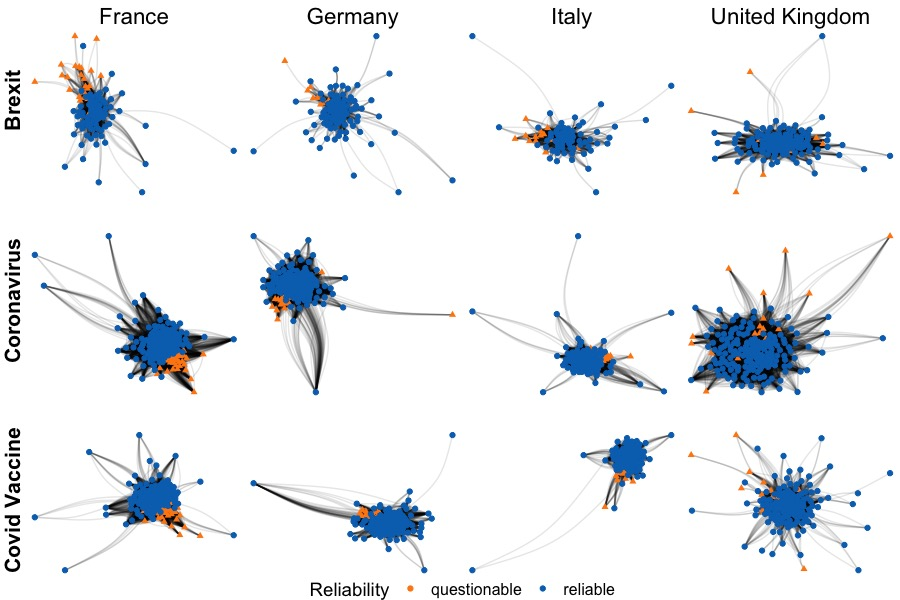

Supplement: S1 Fig — Each news source is represented as a node, and edges represent audiences’ similarity among news outlets. The color and shape of the nodes indicate the classification of the news source, and the thickness of the edges represents the level of similarity of retweeters between two news sources. Each network represents the news outlets’ similarity on one topic for one country. (TIFF) [file pone.0302473.s001.tiff]

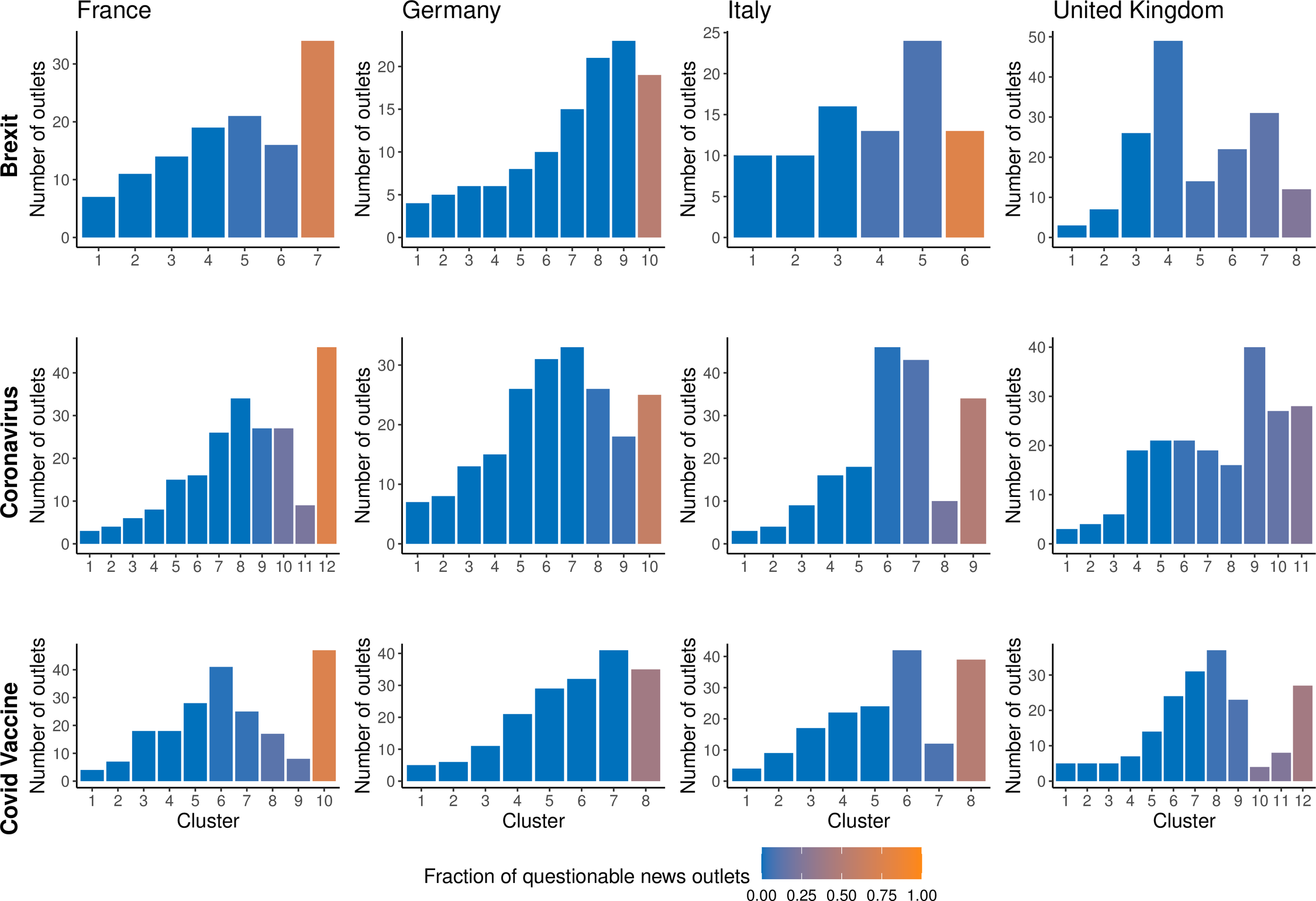

Supplement: S2 Fig — Clusters were found using the Louvain clustering algorithm and sorted based on the percentage of questionable news outlets. The percentage of questionable sources in each cluster is color coded. (TIFF) [file pone.0302473.s002.tiff]

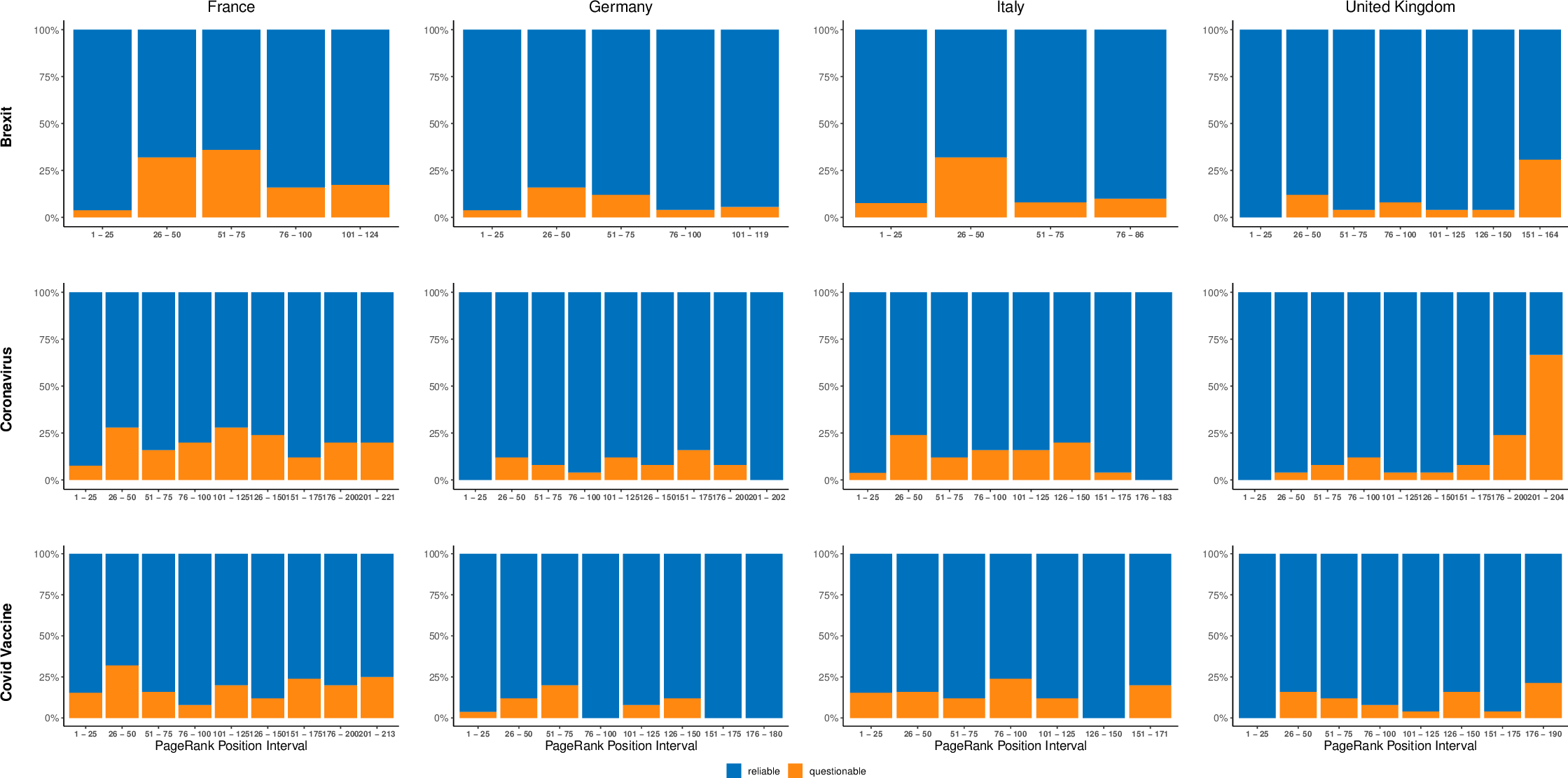

Supplement: S3 Fig — The distribution shows the dominance of reliable sources (blue) over questionable sources (orange). (TIFF) [file pone.0302473.s003.tiff]

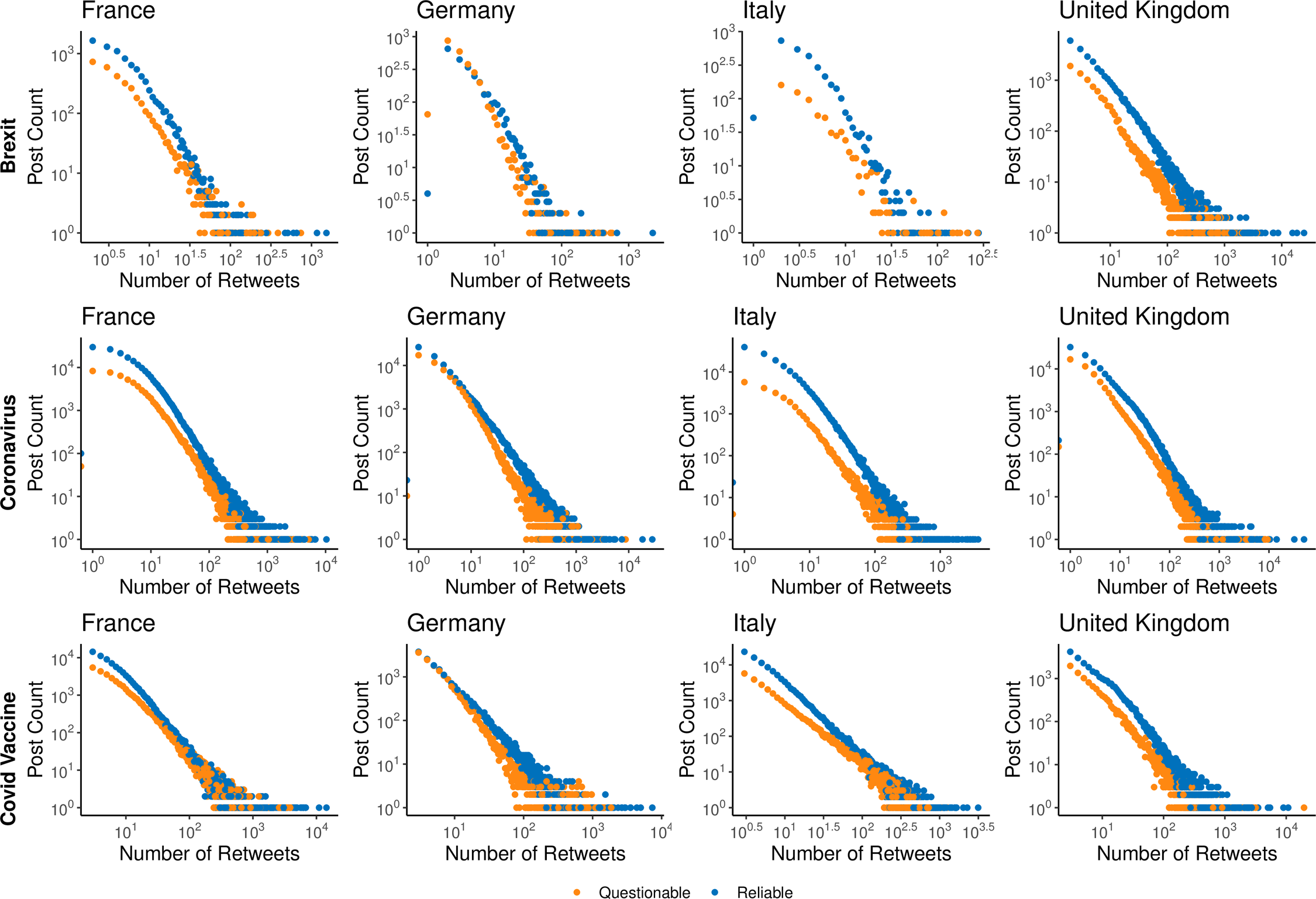

Supplement: S4 Fig — (TIFF) [file pone.0302473.s004.tiff]

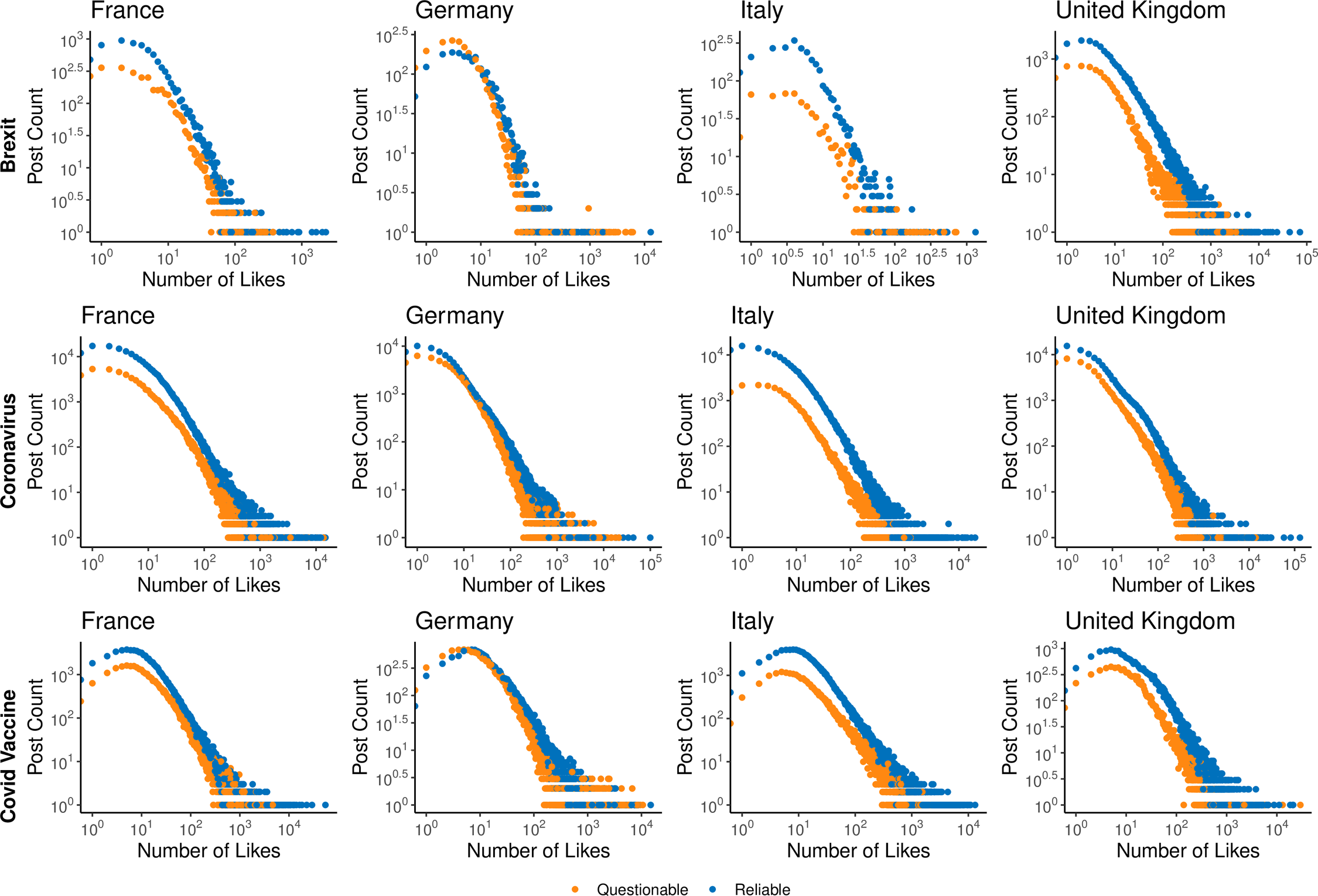

Supplement: S5 Fig — (TIFF) [file pone.0302473.s005.tiff]

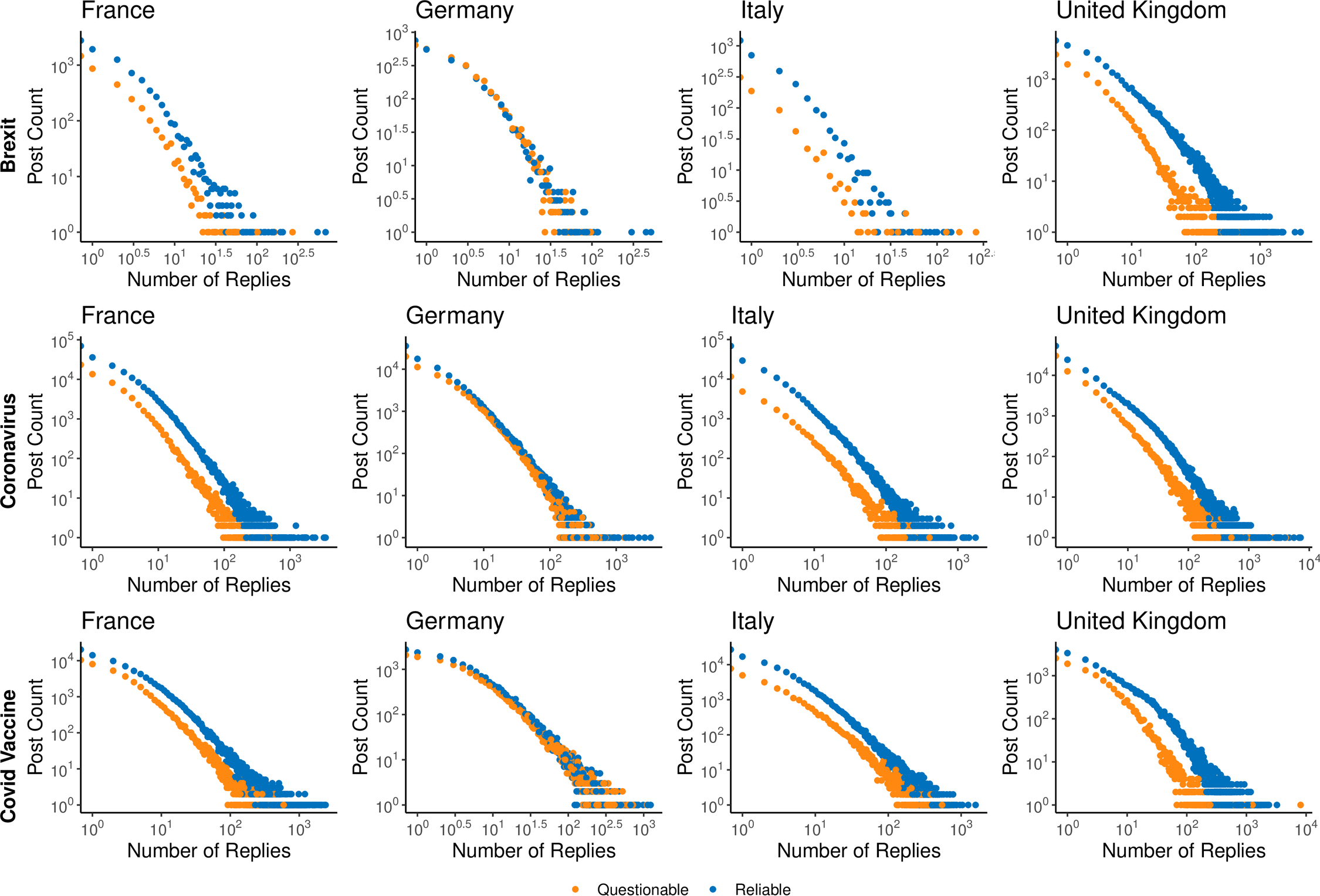

Supplement: S6 Fig — (TIFF) [file pone.0302473.s006.tiff]

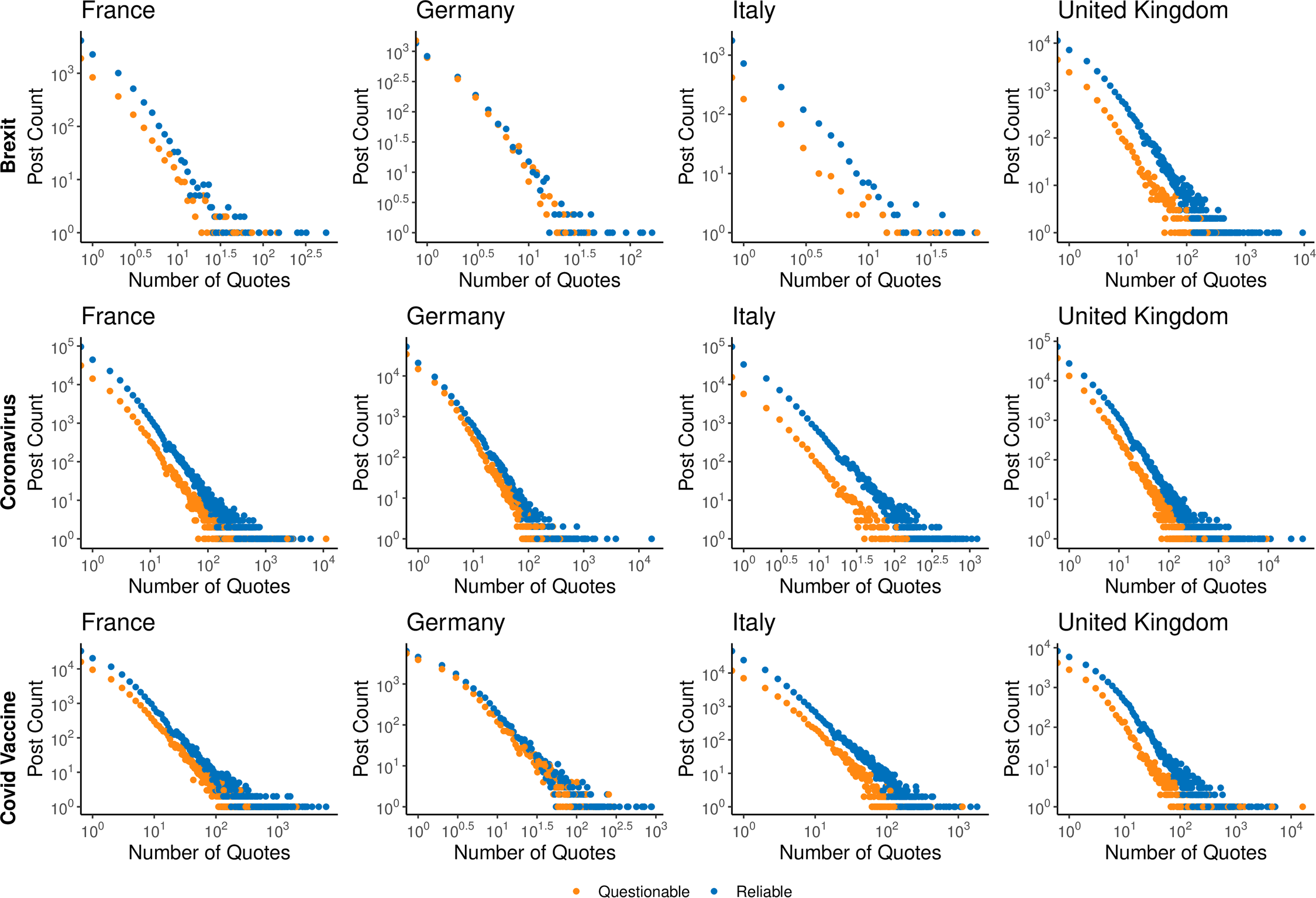

Supplement: S7 Fig — (TIFF) [file pone.0302473.s007.tiff]

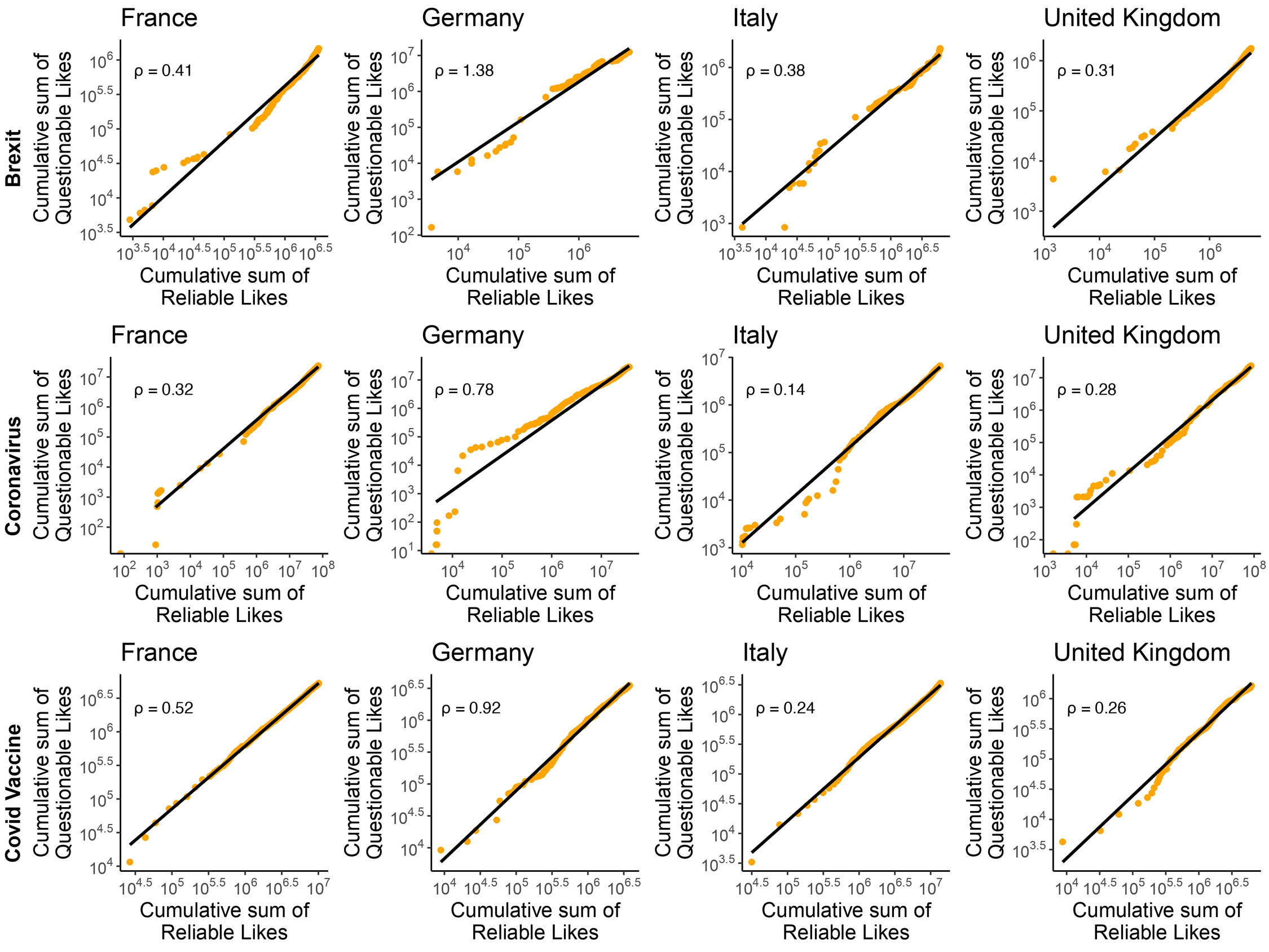

Supplement: S8 Fig — (TIFF) [file pone.0302473.s008.tiff]

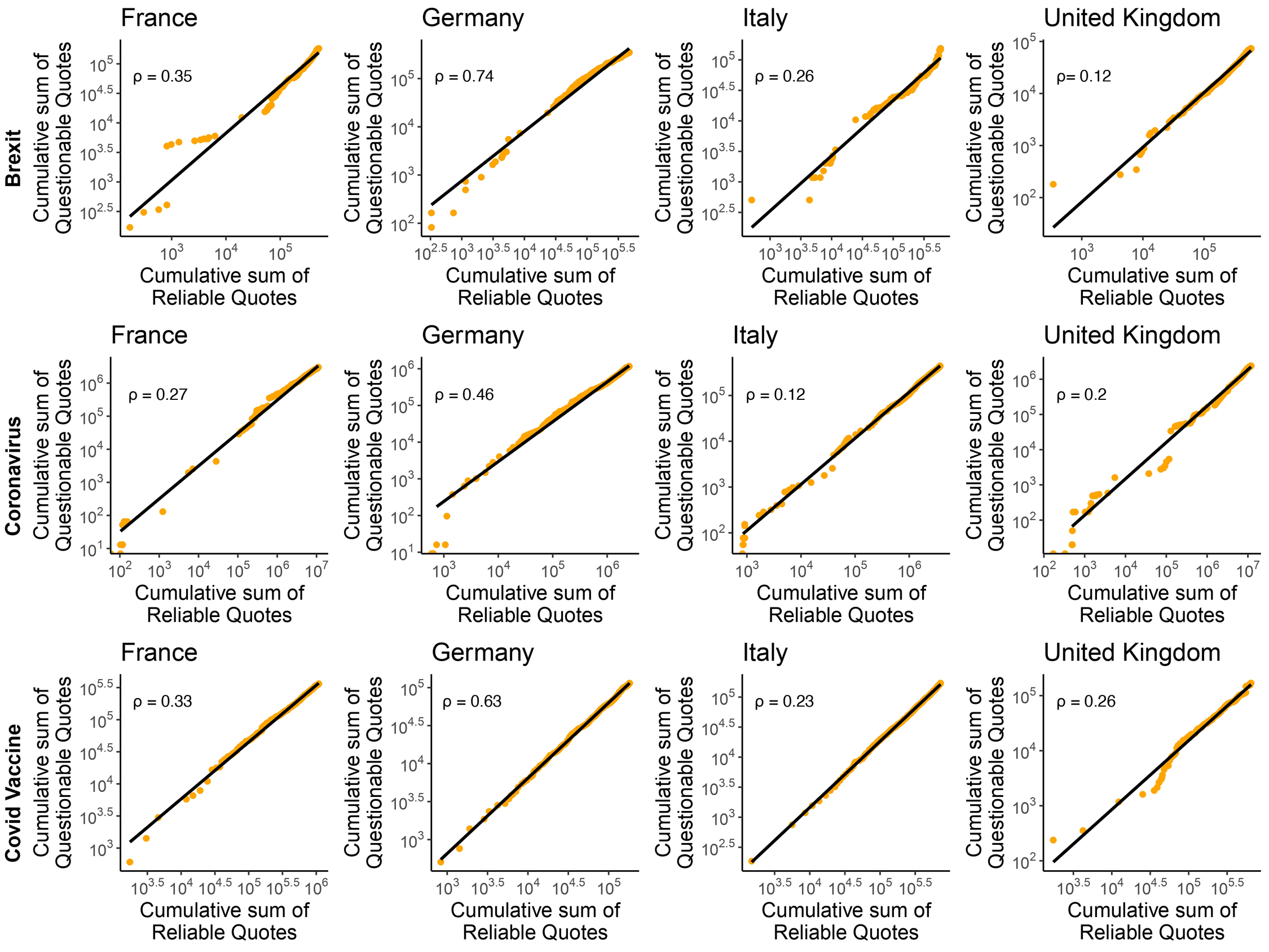

Supplement: S9 Fig — (TIFF) [file pone.0302473.s009.tiff]

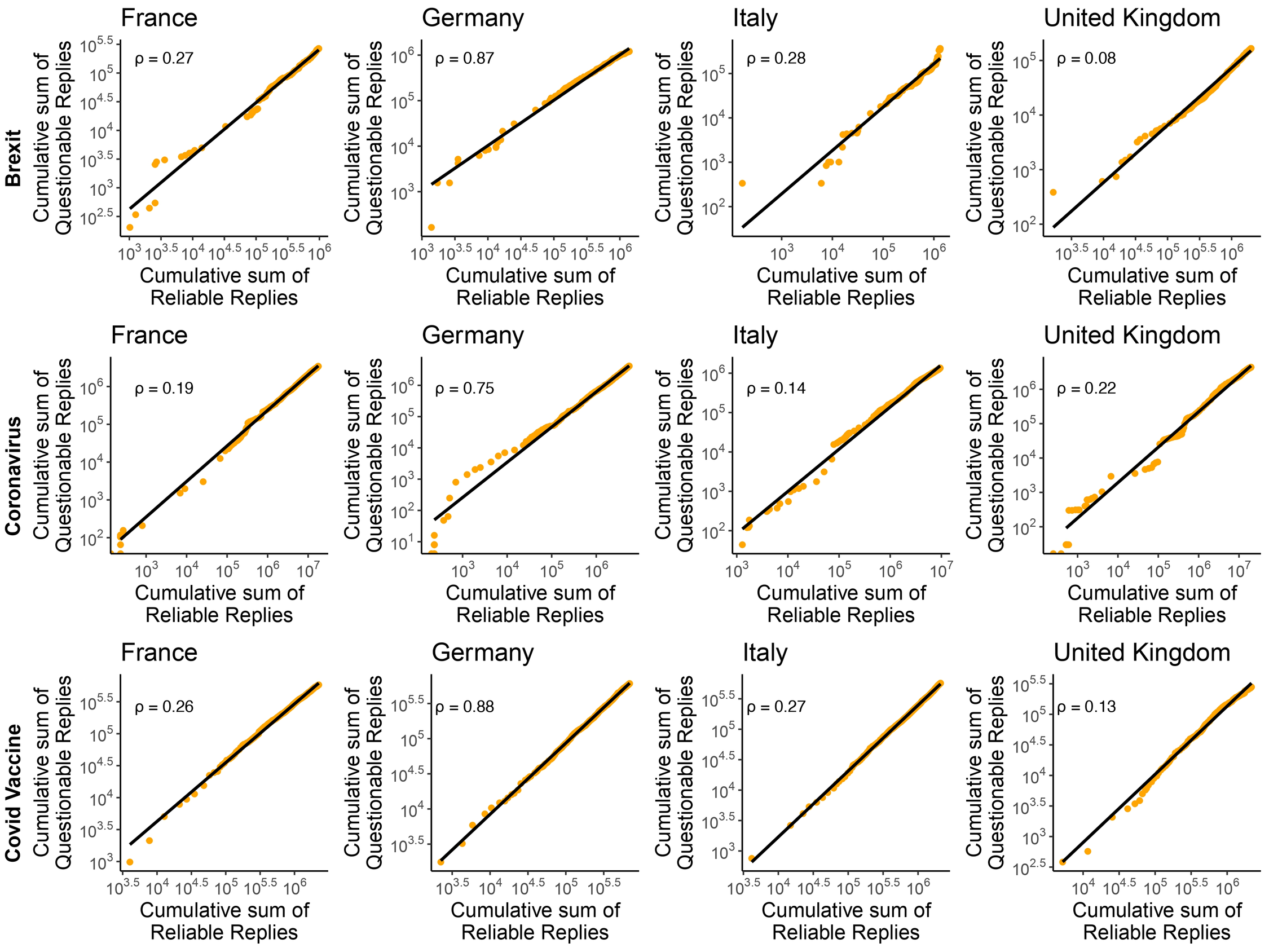

Supplement: S10 Fig — (TIFF) [file pone.0302473.s010.tiff]

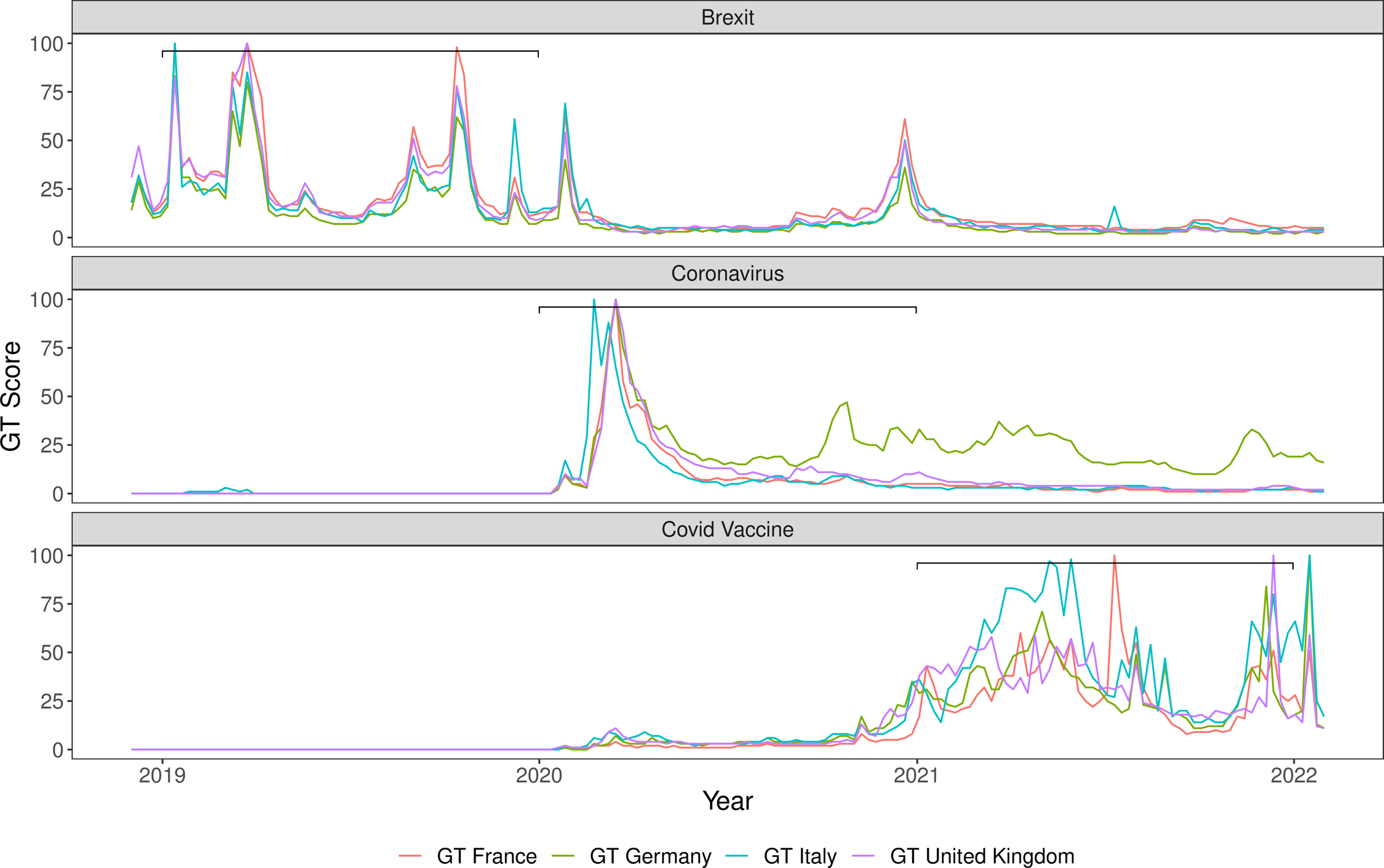

Supplement: S11 Fig — The plots display how search interest for each topic evolved over time, with each row representing one topic. Interest trends reveal that Brexit was most popular in 2019, followed by a sharp decline in 2020 and 2021 with some exceptions at the end of 2020. Coronavirus peaked in early 2020 and declined thereafter, while Covid Vaccine gained momentum in early 2021, reached the maximum in mid-2021, and saw another surge at the end of 2021. Brackets represent the time span taken into account in the analysis for each topic. (TIFF) [file pone.0302473.s011.tiff]
