# Supplementary material for: News and misinformation consumption: A temporal comparison across European countries
Source: PLoS One. 2024 May 8;19(5):e0302473. doi: 10.1371/journal.pone.0302473 (PMC11078435; doi:10.1371/journal.pone.0302473)
Supplement: S1 File — (PDF) [file pone.0302473.s012.pdf]

Table S1 Adjusted assortativity coefficients by topic and country. The table showcases adjusted assortativity coefficients [39] for key topics across France, Germany, Italy, and the UK. These coefficients measure the tendency of nodes to be connected to nodes with similar degrees within each country's topic-based network. Notably, variations across countries highlight distinct patterns of intra-network connectivity for each topic.

**Topic**	**France**	**Germany**	**Italy**	**UK**
Brexit	0.41	0.64	0.48	0.05
Coronavirus	0.25	0.48	0.25	0.18
Covid Vaccine	0.24	0.44	0.21	0.22

Table S2 Edge Density topic and country wise. The table show cases the edge density for each network shown in Fig. 3, indicating the interconnectedness of discussions for each topic by country.

**Topic**	**France**	**Germany**	**Italy**	**UK**
Brexit	0.22	0.15	0.22	0.23
Coronavirus	0.40	0.39	0.37	0.31
Covid Vaccine	0.38	0.31	0.37	0.18

Table S3 Connections between reliable and questionable news sources. The table details the number of connections between reliable and questionable news sources within all the networks, along with the total network edges and the percentage these connections represent.

**Topic**	**Country**	**Connections**	**Total Edges**	**Percentage (%)**
**Brexit**	France	268	1,684	15.92
	Germany	62	1,033	5.99
	Italy	112	801	13.98
	UK	266	3,088	8.62
**Coronavirus**	France	2,389	9,764	24.47
	Germany	567	8,060	7.04
	Italy	1,162	6,230	18.63
	UK	610	6,465	9.43
**Covid Vaccine**	France	2,066	8,546	24.13
	Germany	447	4,944	9.02
	Italy	1,143	5,422	21.08
	UK	410	3,263	12.54

Table S4 Audience count for reliable and questionable news sources. The table presents the audience reach for reliable and questionable news sources, highlighting the different number of consumers for each topic and country.

Topic	Country	Questionable Audience	Reliable Audience
**Brexit**	France	3,808	31,310
	Germany	693	22,122
	Italy	1,860	7,407
	UK	18,074	225,227
**Coronavirus**	France	56,404	449,682
	Germany	14,221	419,530
	Italy	18,624	143,624
	UK	38,204	900,928
**Covid Vaccine**	France	75,953	368,751
	Germany	15,772	157,704
	Italy	27,348	146,502
	UK	23,951	293,756

Table S5 Audience similarity using Jaccard Similarity for reliable and questionable news sources. The table reflects the overlap between the consumers of different pairs of topics (i.e., Brexit - Coronavirus, Brexit - Covid Vaccine, Coronavirus - Covid Vaccine) for both questionable and reliable news sources, respectively. This is done for each country. The low values suggest a minimal overlap in the audiences across the topics. Comparatively, among all three pairs of topics, *"Coronavirus - Covid Vaccine"* has the highest overlap in all countries for both types of sources and particularly for questionable ones.

Questionable				
	**France**	**Germany**	**Italy**	**United Kingdom**
**Brexit-Coronavirus**	0.027	0.022	0.039	0.064
**Brexit-Covid Vaccine**	0.015	0.018	0.021	0.054
**Coronavirus-Covid Vaccine**	0.104	0.162	0.107	0.100
Reliable				
	**France**	**Germany**	**Italy**	**United Kingdom**
**Brexit-Coronavirus**	0.017	0.021	0.015	0.063
**Brexit-Covid Vaccine**	0.018	0.035	0.014	0.070
**Coronavirus-Covid Vaccine**	0.086	0.088	0.087	0.079

Table S6 Coefficients and R^2 of the linear regressions.

Topic	Country	Intercept	Coefficient (ρ)	R^2
Likes				
**Brexit**	France	-149749.62	0.41	0.99
	Germany	1002398.05	1.83	0.96
	Italy	-75787.18	0.38	0.96
	UK	-147194.38	0.31	0.98
**Coronavirus**	France	-206134.61	0.32	0.99
	Germany	-916347.65	0.78	0.98
	Italy	-106666.15	0.14	0.99
	UK	-732903.90	0.28	0.99
**Covid Vaccine**	France	142741.59	0.52	0.99
	Germany	20840.95	0.92	0.99
	Italy	-114257.72	0.24	0.99
	UK	60577.72	0.26	0.98
Replies				
**Brexit**	France	1812.20	0.27	0.99
	Germany	77470.09	0.87	0.99
	Italy	-20301.90	0.28	0.86
	UK	-11296.79	0.08	0.99
**Coronavirus**	France	72290.45	0.19	0.99
	Germany	-102763.32	0.75	0.99
	Italy	42936.78	0.14	0.99
	UK	90964.23	0.22	0.99
**Covid Vaccine**	France	31329.15	0.26	0.99
	Germany	-3914.71	0.88	0.99
	Italy	-18436.29	0.27	0.99
	UK			
Quotes				
**Brexit**	France	-3566.67	0.35	0.99
	Germany	33951.71	0.74	0.98
	Italy	-4289.29	0.26	0.93
	UK	-5394.81	0.12	0.99
**Coronavirus**	France	128424.68	0.27	0.99
	Germany	-14419.62	0.46	0.99
	Italy	2045.06	0.12	0.99
	UK	-81130.22	0.2	0.99
**Covid Vaccine**	France	20083.03	0.33	0.99
	Germany	189.94	0.63	0.99
	Italy	-6376.25	0.23	0.99
	UK	-13767.83	0.26	0.97
Retweets				
**Brexit**	France	-568.73	0.45	0.99
	Germany	209174.96	0.77	0.97
	Italy	-19994.82	0.36	0.97
	UK	-62498.14	0.3	0.98
**Coronavirus**	France	-13707.35	0.35	0.99
	Germany	-151421.99	0.43	0.98
	Italy	-42453.55	0.17	0.99
	UK	-478008.98	0.31	0.99
**Covid Vaccine**	France	16183.82	0.62	0.99
	Germany	-4797.01	0.71	0.99
	Italy	-70371.66	0.43	0.98
	UK	18110.44	0.32	0.99
